# Molecular and histological traits of reduced lysosomal acid lipase activity in the fatty liver

**DOI:** 10.1038/s41419-021-04382-4

**Published:** 2021-11-18

**Authors:** Simone Carotti, Daniele Lettieri-Barbato, Fiorella Piemonte, Sergio Ruggiero, Marco Rosina, Francesca Zalfa, Maria Zingariello, Francesca Arciprete, Francesco Valentini, Maria Francesconi, Jessica D’Amico, Antonio De Vincentis, Andrea Baiocchini, Giuseppe Perrone, Raffaele Antonelli-Incalzi, Sergio Morini, Antonio Picardi, Katia Aquilano, Umberto Vespasiani-Gentilucci

**Affiliations:** 1 Unit of Microscopic and Ultrastructural Anatomy, CIR, University Campus Bio-Medico, Rome, Italy; 2 Department of Biology, University of Rome Tor Vergata, Rome, Italy; 3 Unit of Neuromuscular and Neurodegenerative Diseases, Children’s Hospital and Research Institute “Bambino Gesù”, Rome, Italy; 4 Internal Medicine, Geriatrics and Hepatology Unit, University Campus Bio-Medico, Rome, Italy; 5 Paediatric Unit Sant’Andrea Hospital, Faculty of Medicine and Psychology, “Sapienza” University, Rome, Italy; 6 Laboratory of Pathology of the National Institute for Infectious Diseases, Lazzaro Spallanzani, Rome, Italy; 7 Unit of Anatomical Pathology, University Campus Bio-Medico, Rome, Italy

**Keywords:** Non-alcoholic fatty liver disease, Translational research

## Abstract

Recent studies demonstrated reduced blood lysosomal acid lipase (LAL) activity in patients with nonalcoholic fatty liver disease (NAFLD). We aimed to verify hepatic LAL protein content and activity in in vitro and in vivo models of fat overload and in NAFLD patients. LAL protein content and activity were firstly evaluated in Huh7 cells exposed to high-glucose/high-lipid (HGHL) medium and in the liver of C57BL/6 mice fed with high-fat diet (HFD) for 4 and 8 months. LAL protein was also evaluated by immunohistochemistry in liver biopsies from 87 NAFLD patients and 10 controls, and correlated with hepatic histology. Huh7 cells treated with HGHL medium showed a significant reduction of LAL activity, which was consistent with reduced LAL protein levels by western blotting using an antibody towards the N-term of the enzyme. Conversely, antibodies towards the C-term of the enzyme evidenced LAL accumulation, suggesting a post-translational modification that masks the LAL N-term epitope and affects enzymatic activity. Indeed, we found a high rate of ubiquitination and extra-lysosomal localization of LAL protein in cells treated with HGHL medium. Consistent with these findings, inhibition of proteasome triggered dysfunctional LAL accumulation and affected LAL activity. Accumulation of ubiquitinated/dysfunctional LAL was also found in the liver of HFD fed mice. In NAFLD patients, hepatic levels of non-ubiquitinated/functional LAL were lower than in controls and inversely correlated with disease activity and some of the hallmarks of reduced LAL. Fat overload leads to LAL ubiquitination and impairs its function, possibly reducing hepatic fat disposal and promoting NAFLD activity.

## Introduction

Lysosomal acid lipase (LAL) plays a key role in lipid metabolism by hydrolysing cholesteryl esters and triglycerides in lysosomes [1]. In hepatocytes, LAL has been also shown to help the degradation of intracellular lipids through the lysosomal-autophagy pathway [2, 3]. When LAL activity is strongly reduced or absent, due to homozygous or heterozygous mutations in the *LIPA* gene, cholesteryl esters and triglycerides accumulate prevalently within lysosomes, and several complications arise due to both lipid overload at the target organ level and to dyslipidaemia, secondary to upregulation of endogenous cholesterol production by hydroxyl-methyl-glutaryl coenzyme-a (HMG-CoA) reductase [4]. Indeed, Wolman disease (WD) and cholesteryl ester storage disease (CESD) are characterized by massive microvesicular steatosis rapidly evolving to cirrhosis and liver failure [4], with timing and type of clinical phenotype being strongly dependent on the absent (WD), or minimal residual (CESD), enzyme activity.

Recently, thanks to the availability of a new test on dried blood spot (DBS) [5], which has significantly simplified the determination of LAL activity, LAL function has been evaluated in patients with chronic liver disease, particularly in those with nonalcoholic fatty liver disease (NAFLD) and with cirrhosis due to nonalcoholic steatohepatitis (NASH) [6–10]. In these studies, DBS-determined LAL activity was found to be reduced in adult NAFLD patients with respect to controls, more severely in those with histological NASH and/or post-NASH cirrhosis, suggesting that impaired LAL activity may contribute to the pathophysiology of liver damage. However, concerns about the reliability of the DBS test in the context of liver disease have been raised [11], underscoring the need to verify LAL content and activity directly at the target organ, i.e. hepatic, level. In this regard, the only data available have been provided very recently by Gomaraschi et al., who found a good correlation between DBS-determined and hepatic LAL activity in NAFLD patients [10].

Based on this background, in the present study, we have explored LAL protein content as well as activity both in in vitro (fat-loaded hepatocyte-derived human carcinoma Huh7 cells) and in vivo (mice fed with high-fat diet, HFD) experimental models of NAFLD in order to clarify the mechanisms underlying LAL activity impairment in NAFLD. We found an accumulation of dysfunctional and extra-lysosomal LAL due to ubiquitination at its N-terminal region. These results were confirmed in the liver of a large cohort of patients with histologically diagnosed NAFLD. Specifically, abnormal accumulation of ubiquitinated LAL was observed in patients with histologically diagnosed NAFLD, in whom it was correlated with disease severity and with some of the hallmarks of impaired LAL function, i.e. microvesicular steatosis and lipolysosomes.

## Methods

### Cells and treatments

The hepatocyte-derived human carcinoma cell line Huh7 was obtained from the American Type Culture Collection (Rockville, MD, USA) and cultured in low-glucose Dulbecco’s Modified Eagle’s Medium (DMEM) with 10% fetal bovine serum, 1% penicillin-streptomycin and 1% L-glutamine in a humidified incubator at 37 °C with 5% CO_2_. For treatments, cells were plated at 80% confluence and, after 24 h, culture medium was replaced with complete high-glucose DMEM and 200 μM of BSA-conjugated palmitic acid was added for 2, 4 and 8 days. Huh7 cells were also treated with the proteasome inhibitor MG132 10 μM (Sigma-Aldrich, Milan, Italy) for 3 h.

Huh7 cells were transfected with a vector encoding for human HA-tagged ubiquitin. In particular, Huh7 cells were transfected overnight with 2 μg/ml pDNA and 3 μg PEI reagent/μg pDNA in complete medium. pDNA and PEI reagent (Alfa Aesar Cat. 43896; stock: 1 μg/μl in ddH_2_O) were incubated 25 min at room temperature (RT) in Opti-MEM medium (10% of culture volume).

### Animal model, treatment and biochemical analyses

Mouse experimentation was conducted in accordance with accepted standard of human animal care after the approval by relevant local (The University Animal Welfare Committee—OPBA, Tor Vergata University) and national (Ministry of Health, Legislative Decree No. 26/2014; European Directive 2010/63/UE) committees with authorization n°378/2017-PR.

Adult C57BL/6J (3 months old) male mice (purchased from ENVIGO, Italy) were randomly divided into four groups: mice fed with normal diet (ND: 3.85 kcal/g among which 10% kcal from fat, 20% from protein and 70% from carbohydrate) for 4 months (group 1) or for 8 months (group 2); mice fed with HFD (5.24 kcal/g among which 60% kcal from fat, 20% from protein and 20% from carbohydrate) for 4 months (group 3) or for 8 months (group 4). ND (#D12450B) and HFD (#D12492) were from Research Diets, INC (New Brunswick, NJ, USA). ND contained 38% sugars, 4% saturated fats, 6% unsaturated fats and casein; HFD contained 20% sugars, 54% saturated fats, 6% unsaturated fats and casein. Mice were maintained at 23.0 ± 1.0 °C and 55.0 ± 5.0% relative humidity under a 12/12 h light/dark cycle (lights on at 6.00 a.m., lights off at 6.00 p.m.).

At the end of treatment, mice were sacrificed by cervical dislocation and liver tissue was explanted for the analyses.

### Patients

In all, 87 patients who had undergone liver biopsy for grading and staging of NAFLD at the Hepatology Unit of the University Hospital Campus Bio-Medico of Rome and whose paraffin-embedded liver tissue was available for further analysis, were included in the study. It was confirmed that there was no history of alcoholic intake >20 g/day if woman and >30 g/day if man or of use of drugs known to induce liver damage, and that the following results had been obtained: negative anti-HCV antibodies and HBsAg; antinuclear antibodies (ANA) < 1:80 and negative anti-mitochondrial (AMA), anti-smooth muscle (ASMA) and anti-liver and kidney microsomal (anti-LKM) antibodies; normal transferrin saturation and serum levels of ceruloplasmin and alpha-1 antitrypsin. All patients had signed an informed consent in which the possible use of part of their hepatic tissue for research purposes was specified.

A convenient sample of ten patients with normal transaminases, testing negative for HBsAg and anti-HCV antibodies, and presenting histologically normal liver with less than 5% steatosis was included in the study as the control group for histological and immunohistochemical (IHC) evaluations. They were five cadaveric liver donors and five patients who had undergone liver surgery for metastatic tumours (two colon cancers, two biliary cystadenomas, one gastric cancer, one endocrine tumour). In metastatic patients, liver tissue for immunohistochemistry (IHC) was at least 0.5 cm from the metastatic lesion.

The protocol of the study conformed to the ethical guidelines of the 1975 Declaration of Helsinki and was approved by the Ethics Committee of the University Campus Bio-Medico of Rome.

### Enzymatic activity

LAL activity was evaluated both in Huh7 cells and in mouse liver tissue samples. Cells were lysed in 0.15 mol/l acetate buffer (pH 4.0) and 1% Triton X-100. Lalistat-2 was used as a protease inhibitor. Liver tissue sections (~1 mm) were homogenized in 1% Triton 100X with protease inhibitors and diluted with 0.3 mg/ml of proteins. Reaction mixture contained 4-methylumbelliferone (4-MU, Apollo Scientific, Manchester, UK), cardiolipin (Sigma Aldrich Company Ltd, Dorset, England) and LAL Lalistat-2 (provided by Alexion Pharma, Cheshire, Connecticut, USA). To carry out the experiment, a buffer solution was prepared using 0.15 M of acetate buffer at pH 4.0 and 1.0% of Triton 100X. Then, 14 ml of buffer were stored in water (37 °C) and for substrate buffer solution 1.0 ml (0.5%, w/v) of cardiolipin in methanol and 400 μl (13.3 mM) of 4 MU-palmitate in DMSO were used. Finally, 30 μM Lalistat-2 was prepared each time by diluting 200 μM Lalistat-2 (in DMSO) with distilled water. A 3.2 mm spot was inserted into the well of a 96-microtiter plate (Greiner bio-one, Germany) and diluted in 200 μl of water for 1 h at RT with slight tilting (50*g*). Reactions were performed in duplicate. The reaction mixture contained 40 μl sample and 10 μl of the inhibitor Lalistat-2. After 10 min pre-incubation, 150 μl substrate buffer was added and the plate incubated for 3 h at 37 °C. Reaction was stopped by adding 100 μl (15 mM) HgCl_2_. A 0–2.5 nmol/well 4 MU standard curve was built. The fluorescence intensity was measured using a Multimode plate reader EnSpire (Perkin Elmer, USA) (λ excitation = 355 nm, λ emission = 460 nm). LAL activity was determined by subtracting activity in the inhibited reaction from uninhibited reaction (total lipase) and expressed as nmol/spot/h of 4MU (methylumbelliferone).

### Immunoprecipitation assay

Huh7 cells (100 mm dish) were lysed in 1 ml of RIPA buffer (50 mM Tris–HCl, pH 8.0, 150 mM NaCl, 12 mM deoxycholic acid, 0.5% Nonidet P-40, and protease and phosphatase inhibitors). Then, 1 mg of total proteins was pre-cleared (4 h at 4 °C under gentle mixing) with 30 μl of Agarose Goat IgG, polyclonal-Isotype Control (Abcam, ab104155). The cleared lysate was incubated with 30 μl of Agarose Anti-HA tag antibody (Abcam; ab214758) or isotype control (16 h at 4 °C under gentle mixing). The immunoprecipitate (IP) was collected at 1000*g* for 2 min followed by three washes with RIPA buffer. IP was denatured with 40 μl of 2X Laemmli buffer and 30 μl were loaded on SDS-PAGE.

### Western blot

Animal liver tissue and cells were homogenized in RIPA buffer. To obtain the cytosolic fraction, Huh7 cells (150 cm dish) were resuspended in 3 ml of sucrose buffer (250 mM sucrose, 10 mM Hepes, 1 mM EDTA, pH 7.4) and lysed by 30 strokes through a Potter-Helvenheim homogenizer in ice. Membrane and organelles were separated at 20,000*g* for 20 min at 4 °C. The supernatant (cytosol-enriched fraction) was concentrated 10-fold with 30 K cut-off centrifugal filters (Amicon^®^ Ultra - 2 ml with 30 K Ultracel^®^ membrane; UFC203024; Merck-Millipore). Then, 10 μg proteins were loaded on SDS-PAGE and subjected to western blotting. Nitrocellulose membranes were incubated with anti-heat shock protein-60 (Hsp60) (Abcam, ab46798), anti-TOM20 (Santa Cruz Biotechnology, Sc17764), anti-Vinculin (Santa Cruz Biotechnology, Sc73614), anti-Ubiquitin (Santa Cruz Biotechnology, Sc-8017), anti-cathepsin D (AbD Serotec, Cat 1910-8997), anti-LAMP-1 (NovusBiologicals, NBP2-61619), anti-LAL (Proteintech, 12956-1-AP), anti-LAL (Novus, NBP1-54155) and anti-LAL (Abcam, ab154356) primary antibodies at 1:1000 dilution. Successively, membranes were incubated with the appropriate horseradish peroxidase-conjugated secondary antibodies. Immunoreactive bands were detected by a FluorChem FC3 System (Protein-Simple, San Jose, CA, USA) after incubation of the membranes with ECL Selected Western Blotting Detection Reagent (GE Healthcare, Pittsburgh, PA, USA).

### Liver pathology

Human liver tissue samples were stained with haematoxylin and eosin and Sirius red, and each case was analysed by the same operator with specific expertise in hepatic histopathology. Severity of steatosis (0–3), features of NASH, including necroinflammation (0–3), hepatocellular ballooning (0–2) and fibrosis stage (0–4) were assessed according to the NAFLD clinical research network scoring system [12], and each case was diagnosed as simple steatosis or NASH according to Brunt et al. [13].

#### Immunohistochemical analysis

IHC was performed on sections obtained from mouse and human formalin-fixed tissues embedded in paraffin. Each case was analysed by IHC for LAMP1, a lysosomal marker (NBP1- 61619, 1:50, mouse monoclonal, Novus Biologicals, Abingdon, UK) and two different LAL antibodies (12956-1-AP, 1:100, rabbit polyclonal, Proteintech, Manchester, UK and NBP1-54155, 1:200, rabbit polyclonal, Novus Biologicals, Abingdon, UK). IHC reactions were visualized by DAB as chromogen from DAKO Omnis Envision FLEX /HRP (California, USA). Haematoxylin was used as counterstains in order to visualize nuclei. IHC-stained sections were scanned using the Hamamatsu NanoZoomer 2.0-RS.

LAMP1 was used to visualize vesicles of lysosomal origin (with a LAMP1-positive membrane). The number of large LAMP1-positive vesicles, with a visible lumen, i.e. lipolysosomes, was evaluated in five fields randomly chosen for each case with a magnification of x400. A mean value was therefore derived in each sample for statistical analysis.

The percentage of hepatocytes with microvesicular steatosis in liver tissue (accumulation of small fat droplets with preserved cellular architecture) [14, 15] was evaluated in four fields randomly chosen for each case (magnification x100), and a mean value was derived. Finally, the samples were divided as follows: a <5% of microvesicular steatosis in the samples was graded as class 1, class 2 from 5–10%, class 3 from 11–25% and class 4 from 26–50%.

For valuation of LAL expression, three randomly chosen fields were analysed for each sample (magnification x100). The immunostaining was evaluated using an open-source plugin IHC Profiler for ImageJ [16]. All images analysed were thresholded at the same intensity value. A scale that ranges from 0 to 3 was applied to describe the intensity of staining in each field, where 0 means negative and 3 means strongly positive. The percentage of liver tissue with different score of intensity were recorded for each field. The IHC score was derived as follows: [(3 + %tissue) X3 + (2 + %tissue) X2 + (1 + %tissue) X1]. A mean value was then derived for each sample [17].

#### Lipid content and fluorescence microscopy analysis

The accumulation of lipid droplets in Huh7 cells was analysed using Oil Red O staining. Cells were fixed with 10% formaldehyde, rinsed with 60% isopropanol and stained with freshly prepared Oil Red O working solution (2 mg/ml in 60% 2-propanol; Sigma-Aldrich) for 20 min followed by four washes of 10 min with PBS. Nuclei were counterstained with Hoechst 33342. Oil Red O-derived fluorescence was visualized by means of a fluorescence microscope Zeiss AXIO observer 7 (Zeiss) equipped with Objective A-Plan 5x/0.12 (cod. 421030-9900-000; Zeiss). Neutral lipid content was estimated through Oil Red O elution with 100% 2-propanol and measured by spectrometric analysis at 492 nm.

Immunofluorescent staining was performed on cells and liver mouse sections from paraffin-embedded tissue. Cells were washed with PBS and fixed with 4% paraformaldehyde for 15 min. Cells were permeabilized and blocked with 0.25% TRITON X-100 and 2% normal serum (in PBS) for 20 min an then incubated in 1% normal serum, 0,1% TRITON X-100 (in PBS) with appropriate primary antibodies for 2 h RT at the following concentration: Ubiquitin antibody (Sc-8017, 1:100, mouse monoclonal, Santa Cruz Biotechnology, Heidelberg, Germany) to recognize ubiquitinated proteins, LAMP1 antibody (NBP1-61619, 1:50, mouse monoclonal, Novus Biologicals, Abingdon, UK) and two different LAL antibodies, (12956-1-AP, 1:50, rabbit polyclonal, Proteintech, Manchester, UK and NBP1-54155, 1:50, rabbit polyclonal, Novus Biologicals, Abingdon, UK).

Mouse formalin-fixed tissues embedded in paraffin were incubated with appropriate primary anti-LAL and anti-LAMP1 (ab24170) antibodies for 2 h RT. For each case, secondary Alexa Fluor 488 and/or Alexa Fluor 568-conjugated donkey anti-mouse, anti-rabbit and anti-goat antibodies (Invitrogen, Carlsbad, CA, USA) were added for 30 min (1:200 titre), followed by another extensive washing step in PBS. Nuclear counterstaining was performed using Vectashield mounting medium (Vector Laboratories, Burlingame, CA, USA) containing 4,6-diamidino-2-phenylindole (DAPI). Fluorescent images were collected with a Nikon A1 Confocal Laser Microscope System (Nikon, Tokyo, Japan). Acquisition was carried out using the Imaging Software NIS-Elements (Nikon) and processed with Fiji software (US National Institutes of Health).

### mRNA analysis

For RT-qPCR analysis, total RNA was extracted by using Quick-RNA extraction kit (Zymo Research). RNA (3 μg) was retro transcribed by using M-MLV (Promega, Madison, WI, USA). qPCR was performed in triplicate by using validated qPCR primers (BLAST), Ex TAq qPCR Premix and the Real-Time PCR (Applied Biosystems). LAL mRNA levels were normalized to alpha-fetoprotein mRNA, and the relative mRNA levels were determined through the 2^−ΔΔCt^ method. Primer sequences for qPCR for *LIPA* gene were: sense 5′ –GACCACTCCCGATGCAACTC- 3′ and antisense 5′- GACCGAGTGTTCCTCACCAG- 3′.

In a subgroup constituted by 32 NAFLD patients and six healthy controls for which sufficient paraffin-embedded tissue was still available after histological and IHC examinations, total RNA was extracted using the High Pure FFPET RNA Isolation Kit (Roche) from 10 μm sections. RNA was quantified with the BioPhotometer^®^ D30 (Eppendorf) and the LIPA and collagen type 1 alpha 2 chain (COL1A2) mRNA expression were quantified by Nanostring nCounter Analysis System. This system utilizes a novel digital colour-coded barcode technology that is based on direct multiplexed measurement of gene expression and offers high levels of precision and sensitivity (<1 copy per cell). The technology uses molecular “barcodes” and single molecule imaging to detect and count hundreds of unique transcripts in a single reaction. The results were normalized for the hepatocyte component in each sample by including in the Nanostring panel 3 hepatocyte markers: alpha-fetoprotein (AFP), albumin (ALB) and transthyretin (TTR), as well as for the total cell component with three housekeeping genes: actin-beta (ACTB), tubulin-beta (TUBB) and histone cluster 1H3 family member a (HIST1H3A). Nanostring nCounter analysis was performed following the manufacturer’s protocol (www.nanostring.com). In brief, 100 ng of total RNA was hybridized in solution at 65 °C for 16 h with specific pairs of ~50 base probes for each gene set mRNA. The reporter probe carries the signal; the capture probe allows the complex to be immobilized for data collection. After hybridization, the probe excess was removed, and the probe/target complexes aligned and immobilized in the nCounter Cartridge. Sample cartridges were placed in the digital analyser for data collection. Colour codes on the surface of the cartridge was counted and tabulated for each target molecule. Raw counts for LIPA and COL1A2 mRNAs were normalized (for hepatocyte markers and for housekeeping genes) and analysed using nSolver Analysis Software (nSAS) to obtain their differential expression analysis in NAFLD samples versus control (healthy) samples.

### Statistics

Depending on the parametric or non-parametric distribution, variables are expressed as mean ± SD or median and 25–75% interquartile range (25–75% IR), respectively. Differences were evaluated by the Kruskal–Wallis test. Correlations were carried out by the Spearman’s rank correlation test. A *p* < 0.05 was considered statistically significant. SPSS software (version 22.00; SPSS Inc., Chicago, IL, USA) was used for statistical analyses.

## Results

### LAL activity is reduced in a cellular model of NAFLD

**Fig. 1:**
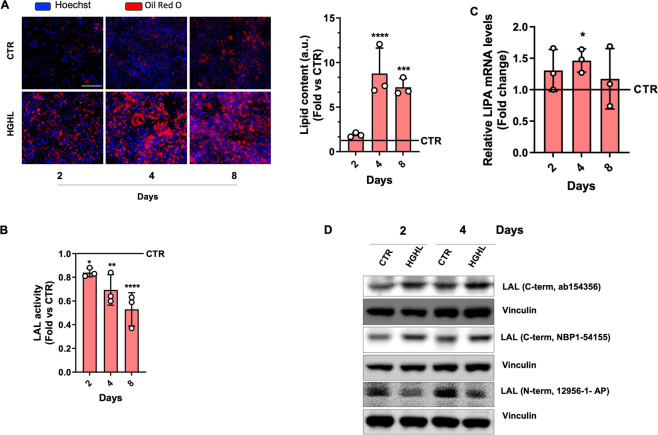
LAL protein content and activity is affected in an in vitro model of NAFLD. Huh7 cells were cultured in high-glucose/high-lipid (HGHL) medium for 2, 4 and 8 days. **A** Oil Red O staining to determine intracellular lipids by fluorescence microscopy (left panel) or by a colorimetric method after Oil Red O elution (right panel). Hoechst 33342 was used to stain nuclei. **B** LAL activity determined by spectrophotometric enzymatic assay. **C** Analysis of *LIPA* mRNA levels by qPCR. **D** Evaluation of LAL protein content through western blot analysis using different antibodies directed against N-term or C-term epitope. Vinculin was used as loading control. Immunoblots are representative of one experiment out of three giving similar results. Data are expressed as mean ± SD (*p < 0.05, **p < 0.01, ***p < 0.001, ^****^p < 0.0001, vs CTR; *n* = 3).

We firstly assessed LAL content and activity in in vitro and in vivo experimental models of NAFLD. In order to trigger lipid overload, human Huh7 hepatocytes were cultured in high-glucose DMEM supplemented with 400 μM palmitic acid (high glucose/high lipid, HGHL) for 2, 4 and 8 days. A significant increase of intracellular lipids was obtained starting from day 4 of treatment (Fig. 1A), which was paralleled by a significant decrease of LAL activity (Fig. 1B). Analysis of mRNA levels showed that *LIPA* gene expression was only slightly increased after 4 days of HGHL treatment (Fig. 1C). Unexpectedly, by western blotting analysis using two different C-terminal LAL antibodies (from 150 to 300 and from 164 to 399 amino acids), a marked accumulation of LAL protein was observed at days 2 and 4 in HGHL cells (Fig. 1D). Conversely, by using an antibody recognizing the N-term region of the protein (from 15 to 200 amino acids), a significant decrease of LAL protein was disclosed in these same cells, which was consistent with the drop of LAL activity (Fig. 1D). Overall, these data suggested that, under HGHL supplementation, in Huh7 cells, a post-translational modification of LAL protein was occurring at the N-term region that masked the antibody epitope, and that such modification determined the accumulation of a ubiquitinated/dysfunctional enzyme.

### The decrease of LAL activity is mediated by ubiquitination

**Fig. 2:**
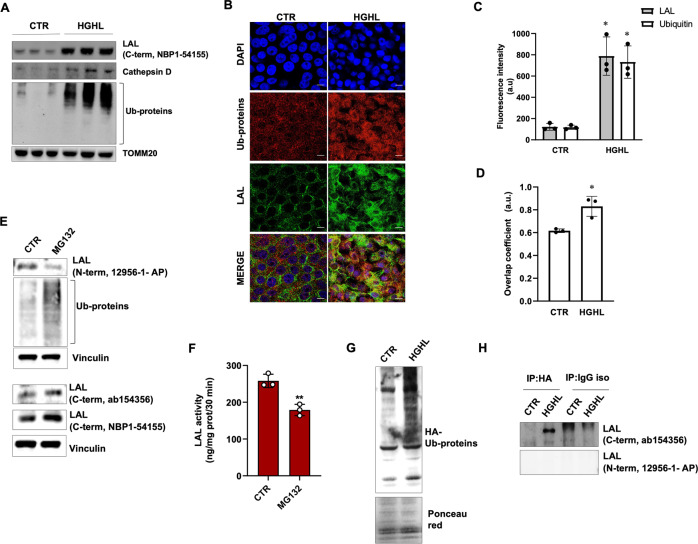
LAL protein undergoes ubiquitination-mediated inactivation in an in vitro model of NAFLD. Huh7 cells were cultured in high-glucose/high-lipid (HGHL) medium for 4 (**A**) or 8 days (**B**–**D**) or with proteasome inhibitor MG132 (10 μM) for 6 h (**E**, **F**). **A** Evaluation of LAL protein, ubiquitinated proteins (Ub-prot) and cathepsin D through western blot analysis. TOMM20 was used as loading control. **B**–**D** Analysis of LAL ubiquitination by confocal microscopy. Ubiquitinated proteins (red) and total LAL protein (individuated by antibody against C-term) (green) were shown (**B**), and their abundance (**C**) and colocalization (**D**) were evaluated. **E** Evaluation of LAL (by different anti-LAL antibodies against C-term or N-term) and ubiquitinated proteins (Ub-prot). Vinculin was used as loading control. **F** LAL activity determined by spectrophotometric enzymatic assay. **G** Representative western blot of Huh7 cells expressing HA-tagged ubiquitin. Anti-HA antibody was used to reveal ubiquitinated proteins under 4 days HGHL treatment. Ponceau red staining was used as loading control. **H** Western blot analysis of LAL by using C-term or N-term antibody on immunoprecipitated HA-ubiquitinated proteins under 4 days HGHL treatment. Immunoblots are representative of one experiment out of three giving similar results. Data are expressed as mean ± SD (*p < 0.05, **p < 0.01, vs CTR; *n* = 3). Scale bar 25 μm.

In order to test whether protein ubiquitination was enhanced in HGHL cells, we carried out a western blot analysis with an anti-ubiquitin antibody. A significant increase of ubiquitinated proteins was observed, which was concurrent with the accumulation of LAL evidenced using the C-terminal LAL antibody, indicating LAL as a possible target of ubiquitination (Fig. 2A). Notably, also the expression of another lysosomal enzyme, i.e. cathepsin, increased under HGHL supplementation, even though at a very minor extent (Fig. 2A). Therefore, we carried out fluorescent immunocytochemical analysis by using the C-term LAL and ubiquitin antibodies. The raise of total LAL and ubiquitinated proteins was confirmed after 4 (data not shown) and 8 days of HGHL treatment through confocal microscopy (Fig. 2B, C), and an increased colocalization between LAL and ubiquitin signals was observed in HGHL cells compared to control cells (Fig. 2D).

To further corroborate these findings, we moved at testing whether LAL protein undergoes ubiquitination and proteasome-mediated degradation in physiological conditions. Huh7 cells were treated for 6 h with the proteasome inhibitor MG132. As evident in Fig. 2E, MG132 was effective in reducing the degradation of ubiquitinated proteins. Notably, western blot analysis carried out with the N-term antibody revealed a decreased LAL content in MG132-treated cells (Fig. 2E), consistent with the hypothesis that ubiquitination masks the epitope recognized by the N-term antibody and that MG132 increased ubiquitinated LAL. By contrast, the two C-term antibodies revealed LAL accumulation upon MG132 treatment (Fig. 2E), suggesting that such antibodies recognized total LAL, i.e. both the ubiquitinated/dysfunctional and the non-ubiquitinated/functional enzyme. Consistently, inhibition of proteasome activity by MG132 led to a significant decrease of LAL activity (Fig. 2F). The specificity of the two antibodies used against the C-term and N-term regions were confirmed in the liver of LAL knockout (KO) mice (Supplementary Fig. 1). Finally, in order to demonstrate the increased ubiquitination of LAL upon HGHL treatment, we overexpressed a HA-ubiquitin and performed an immunoprecipitation assay using an anti-HA antibody. As expected, western blot analysis on total cell lysates revealed an accumulation of HA-ubiquitin proteins in 4 days HGHL-treated cells (Fig. 2G). Immunoprecipitation of HA-ubiquitinated proteins followed by western blot analysis of LAL using the C-term antibody revealed that this protein was ubiquitinated in 4 days HGHL-treated cells (Fig. 2H). Conversely, the staining using the N-term antibody was not able to reveal any signal, confirming that the ubiquitination masks the antibody epitope at N-terminal region of LAL protein (Fig. 2H).

### Extra-lysosomal localization of LAL increases under HGHL treatment

Based on these data, we hypothesized that ubiquitinated/dysfunctional LAL was less able to localize in the lysosomal compartment. Hence, we analysed sub-cellular distribution of LAL by determining the colocalization between LAL and LAMP1, a lysosomal membrane protein. Immunofluorescence assays were performed in HGHL cells by using the C-term LAL and LAMP1 antibodies.

**Fig. 3:**
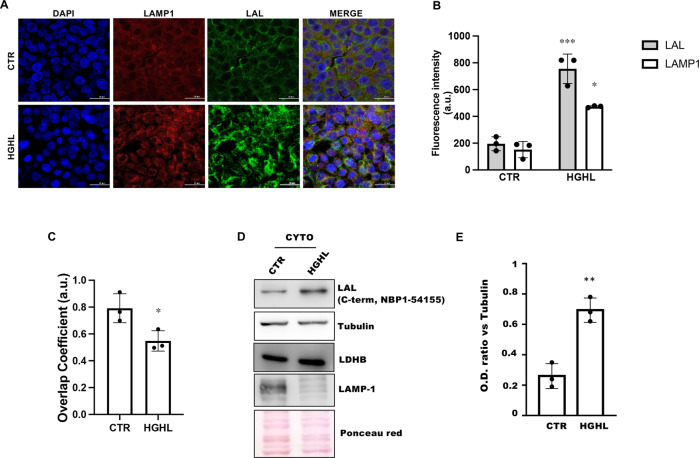
LAL protein accumulates in the extra-lysosomal compartment in an in vitro model of NAFLD. Huh7 cells were cultured in high-glucose/high-lipid (HGHL) medium for 8 days. **A–C** Analysis of LAL localization by confocal microscopy. The lysosomal marker LAMP1 (red) and total LAL protein (individuated by antibody against C-term) (green) were shown (**A**) and their abundance (**B**) and colocalization (**C**) were evaluated. Scale bar 25 μm. **D, E** Western blot analysis of LAL C-terminal domain, LDHB, Tubulin and LAMP-1 in in the cytosolic fraction (**D**) and evaluation of LAL content in the cytosolic fraction by calculating LAL to tubulin ratio through densitometric analysis (**E**). Representative images from one experiment out of three giving similar results are reported. Data are expressed as mean ± SD (*p < 0.05, **p < 0.01 ***p < 0.001, vs CTR; *n* = 3).

The accumulation of LAL was confirmed both at days 4 (data not shown) and 8 in HGHL cells (Fig. 3A, B). This event was accompanied by the increase of the lysosomal marker LAMP1 compared to control cells (Fig. 3A, B), indicating an increase in the lysosomal mass. Interestingly, under HGHL treatment, the colocalization between LAL and LAMP1 was significantly decreased (Fig. 3C), suggesting that LAL accumulates also in the extra-lysosomal compartment. As expected, compared with total LAL, non-ubiquitinated/functional LAL detected with the N-term antibody showed higher colocalization levels with the LAMP1 signal (Supplementary Fig. 2). We isolated cytosolic fraction and determined LAL content through western blot to confirm the data obtained by confocal microscopy. We found that the LAL abundance in the cytosol was increased in line with the idea that upon HGHL treatment LAL accumulates into the cytosol (Fig. 3D, E).

### Ubiquitinated/dysfunctional LAL accumulates in the liver of mice fed HFD

**Fig. 4:**
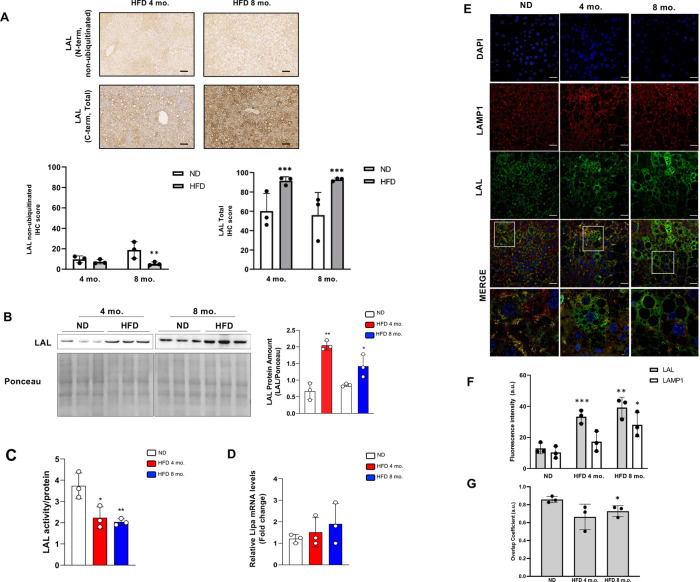
LAL protein content and activity is affected in an in vivo model of NAFLD. C57BL/6J mice were fed for 4 and 8 months with high-fat diet (HFD) or normal diet (ND). **A** Immunohistochemistry analysis of LAL in liver by using different antibodies recognizing total LAL (C-term) or non-ubiquitinated LAL (N-term) and the relative quantification compared to ND. **B** Evaluation of total LAL protein in liver homogenates by western blot analysis (left panel) and relative densitometric analysis (right panel). Ponceau red staining was used as loading control. **C** LAL activity determined by spectrophotometric enzymatic assay. **D** Analysis of LIPA mRNA levels by qPCR. **E–G** Analysis of LAL localization by confocal microscopy. The lysosomal marker LAMP1 (red) and total LAL protein (C-term) (green) were shown (**E**) and their abundance (**F**) and colocalization (**G**) were evaluated. Representative immunohistochemistry and immunofluorescent images are reported that are from one mouse out of 3 for each group. Data are expressed as mean ± SD (*p < 0.05; **p < 0.01, ***p < 0.005, vs ND; *n* = 3 each group). Original magnification X100 (**A**); X400 (**E**), high-power field X600 (**E**). Scale bar 250 μm (**A**); 25 μm (**E**).

We next moved at confirming these findings in an in vivo model of NAFLD consisting in adult C57BL/6J male mice fed with HFD for 4 or 8 months. Mouse fed with ND served as controls. Feeding with HFD was associated with a significant increase in histological steatosis, liver weight, and with elevation of alanine aminotransferase (ALT), glucose and cholesterol levels both at 4 and 8 months [18, 19]. In line with the in vitro data, IHC carried out by using the C-term and N-term antibodies showed an increase of total and a decrease of non-ubiquitinated/functional LAL in the liver of mice fed with HFD with respect to ND, especially at 8 months (Fig. 4A). Consistently, western blot carried out with a C-term antibody revealed that the content of total LAL protein was higher in the liver of HFD fed mice at both time points (Fig. 4B). Moreover, as expected, hepatic LAL activity was significantly decreased in HFD compared to ND mice (Fig. 4C). Notably, hepatic *LIPA* gene expression was similar between HFD and ND mouse groups (Fig. 4D). All together, these results point to the accumulation of ubiquitinated/dysfunctional enzyme in HFD mouse liver.

LAL protein localization was analysed by double-labelling immunofluorescence assays for total LAL and LAMP1 (Fig. 4E). HFD was associated with an increase of the lysosomal marker LAMP1 and of total LAL expression (Fig. 4F), with reduced colocalization between LAMP1 and LAL signals at 4 and 8 months in HFD compared to ND mouse liver (Fig. 4G). These results suggest that ubiquitinated/dysfunctional LAL accumulates in the extra-lysosomal compartment in HFD mouse liver.

### Ubiquitinated/dysfunctional LAL accumulates in the liver of NAFLD patients

Epidemiological, clinical, biochemical and histological characteristics of NAFLD patients included in the study are reported in Table 1. Mean age was just above 50 years and the majority of patients were male (60%). As expected, BMI was in the range of obesity and a high prevalence of diabetes and hypertension was observed. According to the Brunt’s criteria, NASH was diagnosed in 50 patients (57.5%) and all the stages of liver fibrosis were represented.

**Table 1:** Epidemiological, clinical, biochemical and histological characteristics of NAFLD patients.

Variable	Result
Age	51.3 ± 15.3
Sex (M (%)/F (%))	52 (60%)/35 (40%)
BMI (kg/m^2^)	30.4 ± 5.4
AST (IU/l)	48.0 ± 25.7
ALT (IU/l)	88.6 ± 69.3
GGT (IU/l)	83.5 ± 67.3
Glycemia (mg/dl)	102.5 ± 26
Diabetes (no (%)/yes (%))	56 (64.4%)/31 (35.6%)
Hypertension (no (%)/yes (%))	46 (52.9%)/41 (47.1%)
Total cholesterol (mg/dl)	203.8 ± 41.6
HDL cholesterol (mg/dl)	48.6 ± 14.0
Triglycerides (mg/dl)	162.4 ± 94.6
Steatosis (0/1/2/3)	0/32/24/31
Lobular inflammation (0/1/2/3)	11/43/33/0
Ballooning (0/1/2)	11/43/33
NASH (no (%)/yes (%))	37 (42.5%)/50 (57.5%)
Fibrosis (0/1/2/3/4)	14/31/17/15/10

Data are expressed as mean ± standard deviation.

**Fig. 5:**
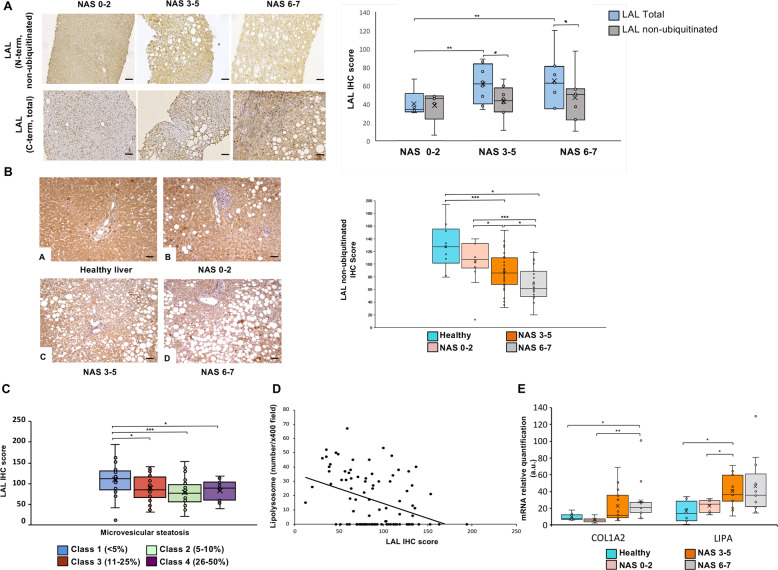
LAL protein content is affected in NAFLD patient. **A** Immunohistochemical (IHC) analysis of LAL in 20 NAFLD patients by using different antibodies recognizing total LAL (C-term) or non-ubiquitinated LAL (N-term) (left panel) and the relative quantification (right panel). **B** Non-ubiquitinated LAL IHC expression was evaluated in healthy subjects (**A**) and NAFLD patients with mild (NAS 0–2) (**B**), moderate (NAS 3–5) (**C**) and severe (NAS 6–7) (**D**) disease activity according to NAS. LAL IHC score was lower in NAFLD patients compared to healthy livers and in moderate-severe NAS compared to mild NAS (right panel). **C**, **D** An inverse correlation between non-ubiquitinated LAL expression and the percentage of microvesicular steatosis and number of lipolysosomes was assessed. **E** *LIPA* mRNA was significantly induced in NAFLD compared to healthy subject, and correlated with NAS (Collagen1A2 was used as marker of fibrosis). Data are expressed as mean ± SD or median and 25–75% interquartile range (25–75% IR), (*p < 0.05; **p < 0.01; ***p < 0.001). Original magnification X100. Scale bar 250 μm.

In a subset of 20 NAFLD patients, non-ubiquitinated/functional and total LAL hepatic content were concurrently evaluated by using the N-term and C-term LAL antibodies, respectively. Total LAL was higher in the livers of patients with NAS score 3–5 and 6–7 compared to those with NAS score 0–2 (Fig. 5A). Furthermore, in patients with NAS score 3–5 and 6–7, the hepatic level of total LAL protein was higher than that of non-ubiquitinated/functional LAL (Fig. 5A), suggesting that the accumulation of ubiquitinated/dysfunctional protein is correlated with NAFLD severity. On the basis of the different trend of IHC expression obtained with C-term and N-term LAL antibodies in this subset of patients, the analysis was extended to a cohort of 87 NAFLD patients, who were studied by IHC with the antibody recognizing the N-term non-ubiquitinated/functional LAL. Non-ubiquitinated/functional LAL protein was significantly lower in NAFLD patients with NAS 3–5 and 6–7 compared to those with NAS 0–2 (Fig. 5B). Non-ubiquitinated/functional LAL was also lower in NAFLD patients with a higher percentage of microvesicular steatosis and with a higher number of fat-engulfed lysosomes, better named lipolysosomes (Fig. 5C, D).

The expression of some relevant genes was finally evaluated by means of Nanostring nCounter Analysis System in a sub-cohort of 32 NAFLD patients and in six healthy controls. *LIPA* gene expression was mildly increased in NAFLD patients compared to healthy controls. Moreover, LIPA and COL1A2 expression showed a trend towards increasing in parallel with the degree of disease activity, with some significant intergroup differences when NAFLD cases were stratified according to NAS score (Fig. 5E).

## Discussion

In the present study, we have comprehensively investigated in in vitro and in vivo experimental models of NAFLD, and in a cohort of histologically diagnosed NAFLD patients, LAL gene (*LIPA*) expression as well as protein content and enzymatic activity. To the best of our knowledge, the obtained results demonstrate for the first time that fat overloading of hepatocytes determines LAL ubiquitination and its reduced localization to the lysosomal compartment, with cytoplasmic accumulation of a dysfunctional enzyme and consequent impairment of its function. In NAFLD patients, the degree of reduction of functional LAL was associated with some of the hallmarks of impaired LAL activity, i.e. microvesicular steatosis and lipolysosomes, and with disease severity in terms of necroinflammatory activity. Altogether, these results point to the possible contribution of an acquired impairment of LAL protein integrity and function to the pathogenesis of NAFLD.

LAL is the enzyme with primary responsibility for hydrolysis of cholesteryl esters and triglycerides within hepatocytes. Thus, in normal conditions, LAL plays a central role in cholesterol homoeostasis by preventing intracellular lipid overload. Hence, reduced LAL activity is causally involved in the increased cholesterol ester storage in lysosomes. Moreover, since the lysosomal-autophagy pathway is strictly involved in hepatic lipid degradation [20], LAL deficiency may impair the efficiency of this process and limit the disposal of fatty acids. Actually, WD and CESD are characterized by severe microvesicular steatosis and rapid progression towards liver failure [4].

Based on this background and thanks to the availability of a new test on DBS to screen for LAL deficiency, blood LAL activity has been recently explored in different cohorts of patients with NAFLD and post-NASH cirrhosis, where it was generally found to be reduced [6–10]. However, the degree of LAL activity reduction observed in these studies was milder than that observed in WD and CESD. Moreover, when investigated [7–10], mutations responsible for a genetically determined impairment of LAL activity were not disclosed, suggesting that LAL function is impaired on an acquired base, likely associated with or determined by hepatic fat overload. However, since most of the lysosomal activities tested in DBS are of leucocyte origin, the reduction of white blood cells typical of advanced liver disease may have affected the results. Actually, a strong correlation between LAL activity and leucocyte count has been observed in all the previous studies in cirrhotic patients [7–9]. Moreover, an even stronger association was found in cirrhotic patients between LAL function and platelet (PLT) count [7–9], and a tight correlation was recently described between LAL activity in DBS and that determined in the PLT fraction of blood [11]. Therefore, in patients with liver disease, verifying LAL expression and activity at the hepatic level have emerged as essential.

In the present study, fat overload in hepatocytes induced a significant impairment of LAL activity. While a mild and transitory increase of *LIPA* gene expression was observed, the results concerning the amount of LAL protein were dichotomous and strictly dependent on the antibody used. In particular, LAL expression was significantly increased when analysed by antibodies recognizing the C-term of the protein, while the opposite was observed with the antibody directed towards the N-term of the enzyme. We therefore hypothesized that LAL ubiquitination was responsible for these findings by masking the epitope on the N-term of the enzyme and we went further demonstrating increased protein ubiquitination, and increased colocalization between LAL and ubiquitin signals, in fat overloaded cells. In agreement with our hypothesis, treatment of Huh7 cells with the proteasome inhibitor MG132 led to a reduction of LAL activity and to the dichotomous findings previously described using the N-term and C-term antibodies, suggesting that LAL turnover is under the control of the ubiquitin/proteasome system also under physiological conditions. This interpretation was further corroborated by the immunoprecipitation assay in HGHL-treated cells followed by western blot analysis for LAL protein using the C-term antibody.

Damaged proteins can be committed to ubiquitination, a post-translational modification of ε-amino group of lysine residues at the protein N-term mediated by the covalent attachment of one or more ubiquitin proteins [21]. Ubiquitinated proteins can be then degraded by the proteasome system [22]. To understand the possible effects of LAL ubiquitination on enzyme function, it is crucial to remember intracellular LAL trafficking, which is typical of lysosomal enzymes. Following signal peptide cleavage in the endoplasmic reticulum (ER), nascent LAL in the ER/Golgi is a 56 kDa pro-peptide, which can be secreted or routed to the lysosome via the mannose-6-phosphate receptor trafficking system [23]. In the lysosome, acidity and proteases determine pro-peptide cleavage to yield an ≈41 kDa mature, active peptide [23–26]. Ubiquitination and targeting to the proteasome may in some way alter the normal trafficking of the enzyme, and this is consistent with the increase of LAL protein in the cytosolic fraction which we observed in conditions of HGHL treatment. Notably, impairing LAL lysosomal localization means affecting its function; indeed, LAL is only active at acidic pH, with maximal activity at pH 4 and very little activity above pH 4.5 [27].

The results obtained in vitro were substantially reproduced in the in vivo experimental model of HFD fed mice. Moreover, the most relevant findings of the in vitro and preclinical model of NAFLD were confirmed in the liver of patients with NAFLD. Specifically, the expression of non-ubiquitinated/functional LAL was inversely associated with the hallmarks of reduced LAL function and, mainly, with NAFLD activity, these results being proof-of-concept and phenotypical translation of all the other experimental findings.

Our results are mostly in agreement with those reported by the only previous study assessing LAL expression and activity in hepatic cells and in the liver of NAFLD patients [10]. Indeed, by using an antibody towards the C-term of the protein, also Gomaraschi et al. observed that the decrease of LAL activity was not correlated with the decrease of LAL protein both in fat-loaded HepG2 cells and in NAFLD patients. In that study, the reduction of enzyme activity was generically attributed to the overload of fatty acids, without further addressing the possible mechanisms. Actually, Gomaraschi et al. did not disclose any difference in hepatic LAL activity between patients with NASH and those with fatty liver, but the sample for this sub-analysis was limited to 24 subjects.

The present study has some limitations. First of all, it was not possible to measure LAL activity in the liver of NAFLD patients, and it was possible to analyse *LIPA* gene expression only in a subgroup of them. Moreover, blood LAL activity was not available from our NAFLD patients, and the correlation with hepatic LAL expression was therefore not possible. Notwithstanding, this study has some important strengths. It was the first to evaluate the possible mechanisms of acquired impairment of LAL activity in dysmetabolic conditions. It includes data from in vitro and preclinical experimental models that were nicely recapitulated in a large cohort of NAFLD patients encompassing all the spectrum of disease, from simple steatosis to NASH and until liver cirrhosis.

In conclusion, the data hereby presented suggest that disturbances in the hepatic distribution and activity of LAL, which is a key enzyme for intracellular fat disposal, may play a role in the pathogenesis of NAFLD. Further studies are awaited in order to confirm our findings, and to verify whether inhibiting LAL ubiquitination or replacing LAL activity may prove beneficial effects in the treatment of NAFLD.

## Supplementary information

Supplementary Figure Legend.

Supplementary Fig. 1.

Supplementary Fig. 2.

## Data Availability

The datasets generated and/or analysed during the current study are available from the corresponding author on reasonable request.
