# Supplementary material for: Molecular and histological traits of reduced lysosomal acid lipase activity in the fatty liver
Source: Cell Death Dis. 2021 Nov 18;12(12):1092. doi: 10.1038/s41419-021-04382-4 (PMC8602623; doi:10.1038/s41419-021-04382-4)
Supplement: Supplementary file 1 — Supplementary Figure Legend. [file 41419_2021_4382_MOESM1_ESM.docx]

**Supplementary Figure 1.** Immunohistochemical analysis in KO and WT mouse liver by using different antibodies against total LAL (C-term) and non-ubiquitinated LAL (N-term). In KO mouse liver a specific immunohistochemical LAL positivity was not individuated. Original magnification X 200. Scale bar 250 μm.

**Supplementary Figure 2. LAL protein decreases in the lysosomal compartment in an *in vitro* model of NAFLD.** Huh7 cells were cultured in high-glucose/high-lipid (HGHL) medium for 8 days. Analysis of LAL localization by confocal microscopy. The lysosomal marker LAMP1 (red) and non-ubiquitinated/functional LAL protein (green) were shown **(A)** and their abundance **(B)** and co-localization **(C)** were evaluated. Representative images from one experiment out of three giving similar results are reported. Data are expressed as means +\- SD (*p<0.05, n=3). Scale bar 25 μm.
