# Supplementary figures and images for: Molecular and histological traits of reduced lysosomal acid lipase activity in the fatty liver
Source: Cell Death Dis. 2021 Nov 18;12(12):1092. doi: 10.1038/s41419-021-04382-4 (PMC8602623; doi:10.1038/s41419-021-04382-4)

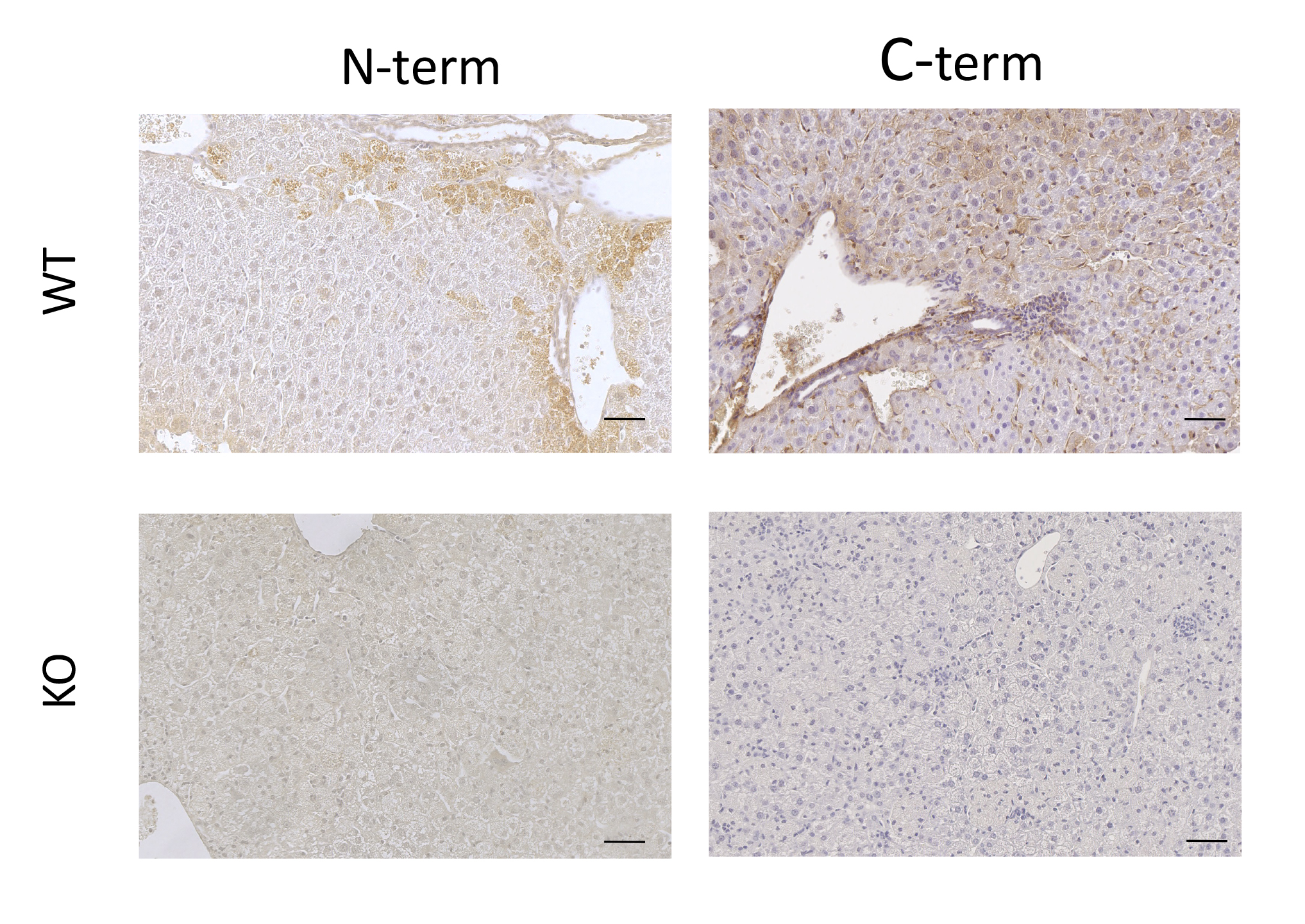

Supplement: Supplementary file 2 — Supplementary Fig. 1. [file 41419_2021_4382_MOESM2_ESM.jpg]

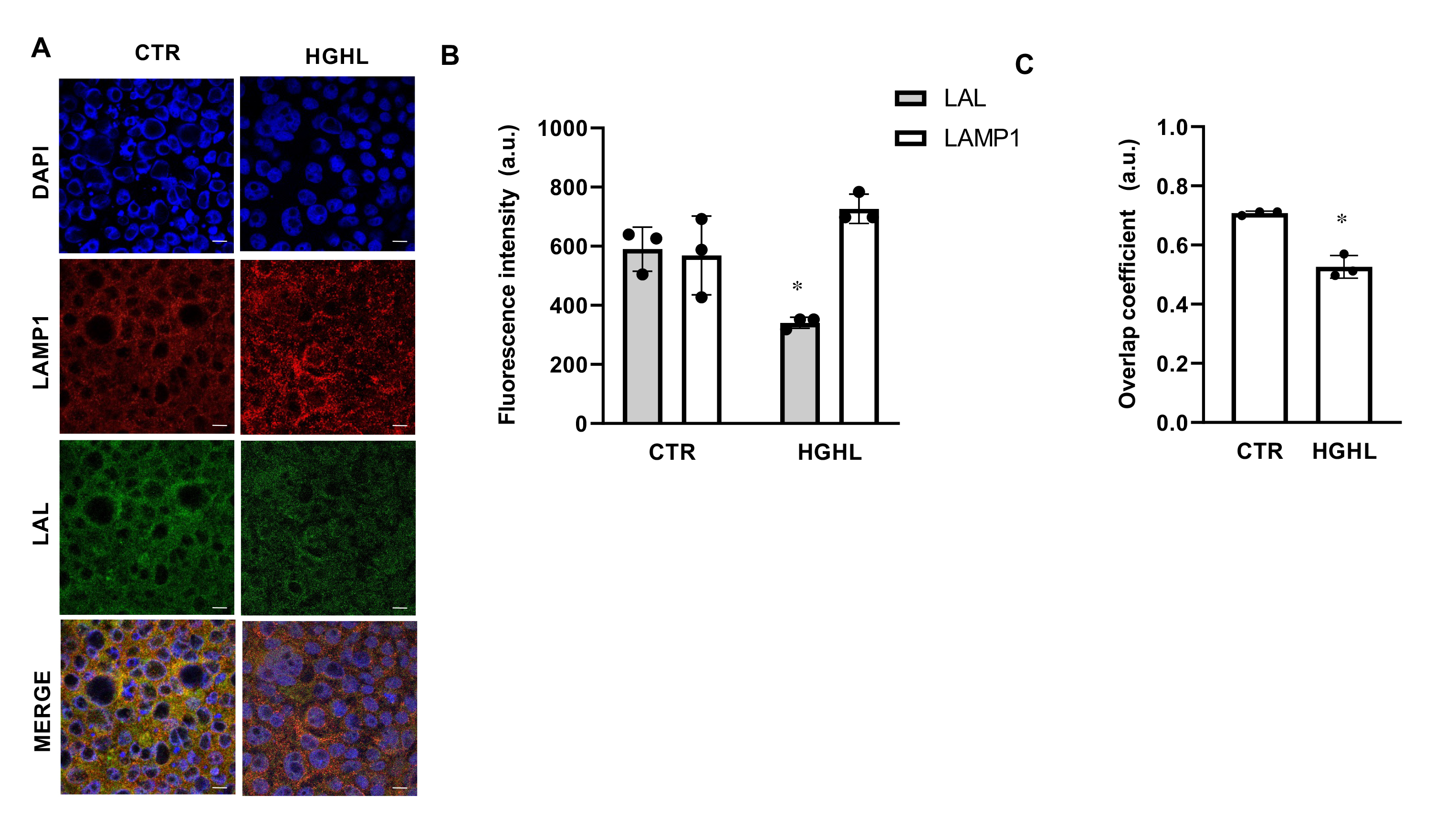

Supplement: Supplementary file 3 — Supplementary Fig. 2. [file 41419_2021_4382_MOESM3_ESM.jpg]
